# Investigating social-contextual determinants of cooperation in incarcerated violent offenders

**DOI:** 10.1038/s41598-018-35450-z

**Published:** 2018-11-21

**Authors:** Jonathan Scheeff, Aiste Jusyte, Michael Schönenberg

**Affiliations:** 1 Department of Clinical Psychology and Psychotherapy, University of Tübingen, Tübingen, Germany; 2 LEAD Graduate School & Research Network, University of Tübingen, Tübingen, Germany

## Abstract

Antisocial and psychopathic personality traits constitute a severe and treatment resistant form of externalizing psychopathology. While deficits in social information processing have been extensively investigated in these individuals, less is known about their capacity for altruism and cooperation. In particular, no studies to date have investigated whether established social-contextual determinants of cooperation, e.g., group affiliation and number of expected interactions, can motivate cooperative behaviour in antisocial individuals. The present study compared cooperative behaviour of incarcerated violent offenders (*N* = 52) and controls (*N* = 46) by using an established social interaction paradigm (Give Some Dilemma) where two players divide monetary units between themselves and the counterpart. Group affiliation (in- vs. out-group) and number of expected interactions (single-trial vs. repeated-trial interactions) were manipulated. Violent offenders as compared to controls shared less monetary units with their counterparts, indicating an overall reduced cooperation. Both groups showed increased cooperation rates towards in-group members and in repeated interactions. Higher psychopathic traits were associated with lower cooperation in single-trial interactions in the violent offender group. Although cooperation was comparably reduced in violent offenders, behaviour in both groups was determined by the number of expected interactions as well as group affiliation, thus providing evidence for equivalent social-contextual determinants.

## Introduction

Antisocial and psychopathic personality tendencies, such as irritability, aggressiveness, ruthlessness and failure to adhere to social rules and norms, as well as a fundamental lack of care for others, constitute severe and treatment resistant forms of externalizing psychopathology. As a consequence, prosocial behaviours such as altruism or cooperation could be assumed to be uncommon for these individuals. Yet, there is anecdotic and factual evidence suggesting that under certain circumstances, these populations also show a willingness for alliance, e.g., forming criminal enterprises. While deficits in social information processing have been extensively investigated in order to explain aggressive and callous behaviour in these populations^1–3^, less is known about their capacity for altruism and cooperation. Understanding to what extent prosocial behaviour is impaired and how it can be reinforced is crucial, considering that this may have direct treatment implications for these severe, costly, and treatment-resistant disorders^4,5^.

Economic games, such as the Dictator Game (DG), the Ultimatum Game (UG), and the Prisoner’s Dilemma (PD), have been widely used to study various facets of prosocial behaviour^6–9^. In the DG, a person (“the dictator”) can freely divide a given amount of resources between himself and a recipient who has no choice but to accept the offer^10^, which is why DG is often employed to measure altruistic behaviour. A mean giving rate of about 30% reported in the DG literature^8^ indicates that average subjects show a generous sharing behaviour at their own expenses. A variation of this paradigm, namely the UG, is constructed in the same manner with the exception that the recipient can deny the proposed share, which results in a loss of gains for both the recipient and the proposer^9^. The UG is therefore thought to reflect the individual’s ability to understand fairness norms in social partners and to adjust the giving behaviour accordingly. This is supported by studies showing that recipients decline offers lower than 30% and that proposers offer a larger proportion (40%) of the pie to the responder in an UG than in the DG^11^. The third paradigm, PD, implements a situation where the participant has the opportunity to either cooperate or defect in a scenario involving another player^7,12^. In a typical paradigm, participants are presented with a scenario in which cooperation leads to greatest collective benefit, while the individual advantage is higher if one person defects and the other does not. In the latter situation, the player who did not defect faces substantial disadvantages. This dilemma between self-interest and collective outcome is assumed to measure cooperation between individuals, with average cooperation rates around 50%^13^ (note that cooperation varies in this paradigm, depending on the used payoff matrix).

There is a large body of research on different aspects of prosocial behaviour in healthy individuals using some variation of these economic games. Recently, these methods have also gained growing attention in clinical psychology where a considerable proportion of disorders are characterized by interactional impairments that are usually difficult to quantify^14,15^. This line of research provides increasing evidence that antisociality and psychopathic traits in particular may be associated with specific deficits in prosocial behaviour. The construct of psychopathy has been introduced to describe a subpopulation within the antisocial spectrum which is characterized by specific traits in the interpersonal (e.g., manipulativeness), affective/cognitive (e.g. callous-unemotional traits), and behavioural domain (antisocial behaviour)^16^. Previous research which employed the DG showed associations between psychopathic traits and attenuated sharing behaviour in both community and incarcerated samples^17–19^. However, decreased proposals in the UG have not been related to psychopathic traits^18,20^, suggesting more sophisticated behaviour when unfair shares can be punished compared to the DG. Studies that investigated cooperative behaviour by employing the PD reported less cooperation in community samples with elevated antisocial behaviour and psychopathic traits^17,20–23^, while the findings for incarcerated samples are less consistent^24,25^. In summary, previous research suggests that while psychopathic and antisocial individuals show attenuated altruism, the responsiveness to descriptive norms appears to be intact, which indicates an understanding of social norms and an ability to adapt their behaviour in order to maximize personal gains. However, cooperation remains an understudied phenomenon in these populations and the psychological mechanisms that govern the decision to cooperate are poorly understood.

The frequency of interactions and the identity of social partners are two important determinants for everyday cooperation. These characteristics of social interactions, namely the affiliation of interaction partners and the number of expected interactions, represent established psychological factors that have been shown to motivate cooperative behaviour^26,27^. In-group members have been known to be favoured over out-group members^28^ and cooperation increases for repeated as compared to single interactions due to potential reciprocity^7^. Further, individuals show an even stronger in-group favouritism when their own outcomes depend on their partner’s behaviour^27^. First attempts to bridge these psychological concepts with antisocial characteristics demonstrate an enhanced in-group favouritism for students with elevated psychopathic traits^29,30^. Further, the absence of cues about future interactions predicted non-cooperation in a PD for students scoring high on psychopathy^23^. However, similar studies in clinical or incarcerated samples are pending.

To investigate cooperative behaviour in antisocial individuals and to test the role of group affiliation and the number of expected interactions in motivating cooperation, we used the Give Some Dilemma (GSD) paradigm^31,32^ in a sample of incarcerated violent offenders and control participants. In this task, participants had an amount of 10 monetary units (MUs) which they could divide between themselves and a counterpart. Group affiliation between the participant and the counterpart (in-group vs. out-group) was established using the minimal group paradigm prior to the experiment^33^. To test for the effects of the number of expected interactions, we manipulated the number interactions with the same counterpart (single-trial interactions vs. repeated-trial interactions with the same counterpart) during GSD, resulting in 60 trials in total (20 single-trial interactions and a total of 40 trials in the repeated interaction condition). Based on the previously reported literature we predicted that:

1. Violent offenders would show generally reduced cooperation, i.e., share less MUs in GSD, when compared to control participants.
2. Cooperation would be modulated by the group affiliation (increased cooperation with in-group members compared to out-group members) and the number expected interactions with the same person (higher cooperation rates in repeated-trial compared to single-trial interactions) in both groups, but to a larger extent in violent offenders.
3. Higher psychopathic traits would be associated with lower cooperation rates, in particular for single-trial interactions and for interactions with out-group members.

## Results

### Participant characteristics

Six control participants were excluded from data analysis due to fully or partially meeting criteria for antisocial personality disorder, resulting in a final sample of 52 violent offenders and 46 controls. In the violent offender group, 35 individuals (67.3%) fulfilled the antisocial personality disorder diagnosis. Twelve violent offenders fulfilled criteria for comorbid substance dependence, one fulfilled criteria for comorbid obsessive-compulsive disorder and seven fulfilled criteria for lifetime major depression episode. Violent offenders were convicted for violent crimes such as aggravated battery, first degree murder, kidnapping, robbery, assault, or threat.

Table 1 displays demographic and clinical sample description for both violent offender and control group. The groups did not differ in terms of years of education and intelligence (*Wiener Matrizen Test* 2, WMT)^34,35^, but violent offenders were slightly older than control participants. Compared to the controls, violent offenders showed higher Buss-Perry Aggression Questionnaire (BPAQ)^36^ scores for all subscales and the total score, except for the ‘hostility subscale’. Elevated scores in violent offenders for the Hare Self-Report Psychopathy Scale III (SRPS)^37^ were observed for the subscale ‘criminal tendencies’ and the total score.

**Table 1:** Demographic and clinical sample description.

	VO (*N* = 52)	CTL (*N* = 46)	Statistics
Demographics			
Age	38.17 (10.04)	33.15 (10.53)	*t*(96) = 2.41, *p* = 0.018
Education (years)	9.63 (1.28)	9.85 (0.63)	*t*(96) = −1.06, *p* = 0.292
WMT score	7.35 (3.87)	8.76 (3.77)	*t*(96) = −1.83, *p* = 0.071
BPAQ			
Physical aggression	22.52 (7.97)	18.80 (6.28)	*t*(96) = 2.54, *p* = 0.013
Verbal aggression	15.94 (3.80)	14.57 (2.86)	*t*(96) = 2.00, *p* = 0.048
Anger	16.69 (5.64)	13.87 (3.98)	*t*(96) = 2.83, *p* = 0.006
Hostility	23.13 (6.56)	21.26 (5.46)	*t*(96) = 1.52, *p* = 0.131
Total score	78.29 (19.87)	68.50 (14.32)	*t*(96) = 2.77, *p* = 0.007
SRPS			
Interpersonal manipulation	2.61 (0.53)	2.51 (0.38)	*t*(96) = 1.02, *p* = 0.308
Callous affect	2.45 (0.58)	2.33 (0.40)	*t*(96) = 1.13, *p* = 0.260
Erratic lifestyle	3.01 (0.60)	2.84 (0.59)	*t*(96) = 1.38, *p* = 0.170
Criminal tendencies	2.89 (0.73)	1.80 (0.51)	*t*(96) = 8.47, *p* < 0.001
Total score	2.74 (0.52)	2.37 (0.35)	*t*(96) = 4.06, *p* < 0.001

*Note*. VO, violent offender group; CTL, control group; WMT, Wiener Matritzen Test 2; BPAQ, Buss-Perry Aggression Questionnaire; SRPS, Hare Self-Report Psychopathy Scale. The data presented in the table refers to means and standard deviations for each measure (in parentheses).

### Cooperative Behaviour

**Figure 1:**
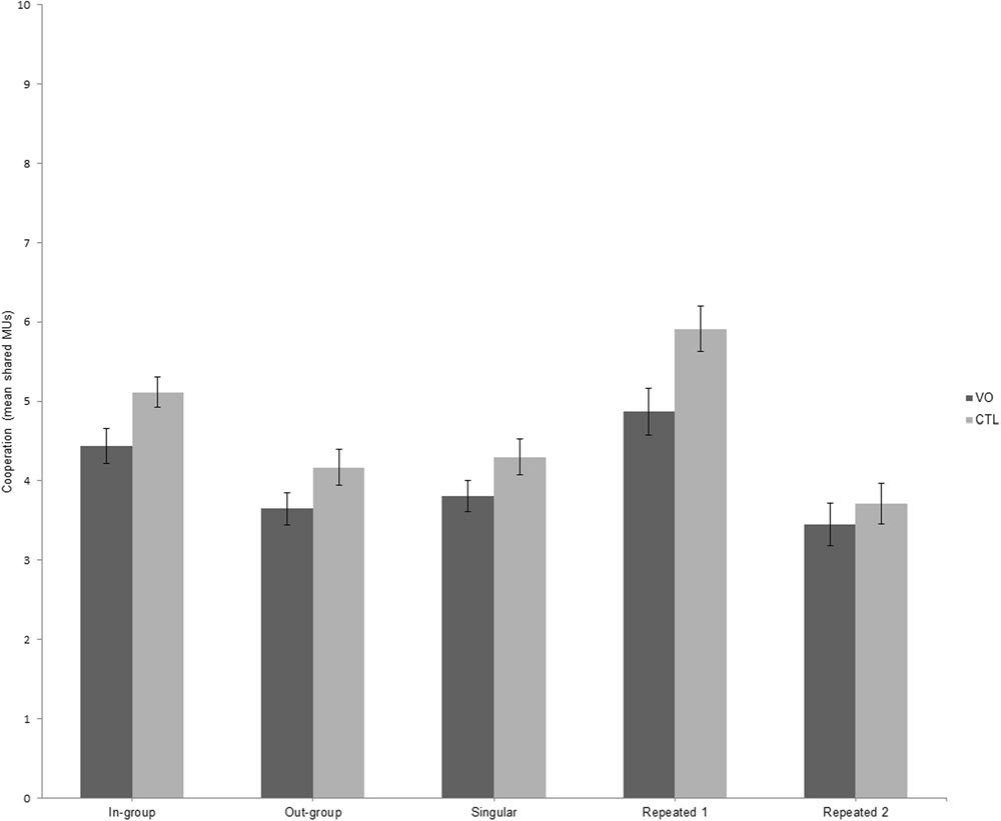
Cooperative behaviour in the Give Some Dilemma as function of shared monetary units (MUs) in violent offenders (VO, *n* = 52) and healthy controls (CTL, *n* = 46); dependent on group affiliation of the other player and number of expected interactions. Singular represents the single-trial interaction condition, Repeated 1 represents the first interaction in the repeated-trial interaction and Repeated 2 represents the second interaction in this block. Error bars indicate standard error from mean.

We computed a 2 (group affiliation: in- vs. out-group) × 3 (number of expected interactions: single-trial interaction vs. first repeated-trial interaction vs. second repeated-trial interaction) repeated measures ANOVA with experimental group (violent offender vs. control) as between-subject variable and shared MUs in the GSD as dependent measure. There was a significant main effect of group (*F*(1,96) = 4.620, *p* = 0.034, *η*_*p*_² = 0.046), indicating lower shared MUs in violent offenders (*M* = 4.34, *SD* = 1.46) compared to healthy controls (*M* = 5.11, *SD* = 1.39). Further, we found a significant main effect of group affiliation (*F*(1,96) = 60.03, *p* < 0.001, *η*_*p*_² = 0.385) which revealed higher sharing behaviour with in-group (*M* = 5.16, *SD* = 1.59) vs. out-group members (*M* = 4.25, *SD* = 1.57) and a main effect for number of expected interactions (*F*(1,96) = 41.29, *p* < 0.001, *η*_*p*_² = 0.301) indicating more shared MUs in the first repeated-trial interactions (*M* = 5.36, *SD* = 2.09) compared to single-trial (*M* = 4.04, *SD* = 1.51) and the second repeated-trial interactions (*M* = 3.57, *SD* = 1.85; see Fig. 1). No other interaction effects reached significance (*p*s > 0.1). These findings show that the experimental manipulations of group affiliation and expected number of interactions were successfully implemented and what that while the violent offenders shared overall less MUs, both groups showed elevated levels of cooperation in the first interaction of the repeated setting and with in-group members.

### Influence of psychopathic traits

We found a significant negative correlation between the SRPS subscale ‘criminal tendencies’ as well as a trend toward negative correlations between the SRPS scales ‘erratic lifestyle’ and the total score with cooperation in single-trial interactions for the violent offender group (Table 2). Further, there was trend for a correlation between the SRPS subscale ‘callous affect’ and cooperation with out-group members for the control group. However, these associations were no longer significant after Bonferroni correction.

**Table 2:** Correlations between sharing behaviour and diagnostic measures for violent offenders (N = 52) and controls (N = 46).

	Single	Repeated	In-group	Out-group	SRPS-IM	SRPS-CA	SRPS-EL	SRPS-CT	SRPS-T
Single	—	0.34*	0.66***	0.74***	−0.23	−0.06	−0.27(*)	−0.30*	−0.26(*)
Repeated	0.23	—	0.87***	0.80***	0.05	−0.02	−0.14	−0.05	−0.05
In-group	0.60***	0.79***	—	0.78***	−0.10	−0.07	−0.22	−0.14	−0.15
Out-group	0.71***	0.73***	0.64***	−	0.04	−0.02	−0.23	−0.21	−0.16
SRPS-IM	−0.08	0.18	0.22	−0.06	—	0.69***	0.67***	0.62***	0.86***
SRPS-CA	−0.05	−0.16	0.02	−0.26(*)	0.51***	−	0.55***	0.54***	0.80***
SRPS-EL	0.12	0.22	0.23	0.17	0.32*	0.37*	—	0.77***	0.88***
SRPS-CT	−0.02	−0.13	−0.04	−0.13	0.23	0.29(*)	0.54***	—	0.88***
SRPS-T	0.01	0.05	0.15	−0.07	0.64***	0.69***	0.82***	0.75***	—

*Note*. Single, shared monetary units (MUs) in single-trial interactions; Repeated, shared MUs in repeated-trial interactions, In-group, shared MUs with in-group counterparts, Out-group, shared MUs with out-group counterparts, SRP-IM, Hare Self-Report Psychopathy Scale (SRP) interpersonal manipulation subscale; SRPS-CA; SRPS callous affect subscale; SRPS-EL, SRPS erratic lifestyle subscale; SRPS-CT, SRPS criminal tendencies subscale; SRPS-T, SRPS total score.

The data represented in the table refers to bivariate correlations between the indicated measures for the violent offenders (top) and controls (bottom). ***p < 0.001, **p < 0.01, *p < 0.05, (*)*p* < 0.10.

### Post-hoc robustness check: Development of cooperation behaviour

We performed additional post-hoc analyses to describe the development of cooperation over the course of the repeated-trial interaction block (see Supplementary Figs S1 and S2). There is literature showing that individuals change their cooperation behaviour as they receive feedback on their choices^38^ which is why we performed these post-hoc analyses to see if this was the case in the repeated-trial interaction condition in the GSD. Regression analyses were computed for the cooperation rates in the first interaction for each group, showing an increase of cooperation over time for controls (*b* = 0.046, *t*(96) = 6.10, *p* < 0.001, *R*^2^ = 0.038, *F*(1, 918) = 37.14, *p* < 0.001) as well as for the violent offenders (*b* = 0.033, *t*(96) = 4.75, *p* < 0.001, *R*^2^ = 0.020, *F*(1, 1038) = 22.54, *p* < 0.001), see Supplementary Fig. S1. This shows that both groups exhibited a slight increase for the first repeated-trial interaction. For the second repeated-trial interaction, the regression analyses showed no change in cooperative behaviour over time, neither for controls (*b* = −0.005, *t*(96) = −0.72, *p* = 0.475, *R*^2^ = −0.001, *F*(1, 918) = 0.5105, *p* = 0.475) nor for the violent offenders (*b* = 0.005, *t*(96) = 0.69, *p* = 0.492, *R*^2^ = −0.001, *F*(1, 1038) = 0.4724, *p* = 0.492), see Supplementary Fig. S2. Thus, cooperation was stable for the second interaction in the reapeated-intercation condition for both groups, despite a lack of a subsequent interaction.

## Discussion

The present study aimed to investigate for the first time cooperative behaviour and its’ social-contextual determinants in a population of incarcerated violent offenders using the GSD. We were interested whether violent offenders’ sharing behaviour could be influenced by established social-contextual determinants of cooperative behaviour, i.e., the number of expected interactions and group affiliation. Further, we aimed to investigate psychopathy as a potential moderator for these determinants. The results can be summarized as follows: 1) In line with Hypothesis 1, as compared to controls, the violent offenders shared less MUs with their counterparts, indicating an overall reduced cooperation. 2) In partial support of Hypothesis 2, violent offenders as well as controls showed increased cooperation rates towards in-group members and in repeated-trial interactions. However, these relationships were equivalent in both groups and therefore did not support the assumption of a stronger response to the determinants in violent offenders. 3) In accordance with Hypothesis 3, higher psychopathic traits were associated with lower cooperation in single-trial interactions in the violent offender group. Contrary to this hypothesis, there was no relationship between psychopathic traits and group affiliation in either group, or the number of expected interactions in the control group. In sum, our results show that despite the generally reduced willingness to cooperate, the violent offenders were able to adapt their sharing behaviour to the task demands in the same way as control participants. Importantly, cooperation in violent offenders was determined by the number of expected interactions as well as group affiliation to the same extent as in healthy controls, thus providing evidence for the existence of equivalent psychological mediators. Further, our findings indicate that psychopathic traits in violent offenders are associated with both, more strategic and profit-maximizing behaviour.

Only two studies to date investigated cooperation in incarcerated individuals as compared to healthy controls using the PD paradigm. While one study reported no differences between inmates and control participants^25^, the other demonstrated lower cooperation rates for incarcerated individuals^24^. Together with the results of the present study, the inconsistent findings across the studies warrant some comment. Similar to the study of Mokros *et al*.^24^, we examined male incarcerated individuals by using a repetitive paradigm, while Khadjavi and Lange^25^ investigated female prisoners with a one-shot and two-trial paradigm. Therefore, inconsistent findings could be attributed to task characteristics (single vs. repeated interaction) or the gender of the participants, as women have been reported to cooperate more in initial trials than men, but this difference disappears as the interaction continues^39^. Further research addressing gender specific differences in incarcerated populations is needed. Nevertheless, the current findings add to an existing body of literature documenting lower rates of sharing behaviour in clinical groups as well as community samples with antisocial and psychopathic traits using other economic games^17,18,20–23^.

To our knowledge, this study is the first to investigate social-contextual determinants of cooperation, i.e., the number of expected interactions and group affiliation, in incarcerated offenders. With regard to the expected number of interactions, our results show that the anticipated frequency of interactions enhances cooperation in both violent offenders and controls, irrespective of the co-player’s affiliation with the in- or the out-group. This replicates the findings previously reported for healthy participants^40^ and extends these results to a clinical population. Our experimental design featured aspects from both, the DG (absence of consequences for sharing behaviour as in the single-trial interaction condition) and the UG (possibility for punishment as in the repeated interaction condition) where analogous results have been reported, in particular lower sharing behaviour in the DG^17–19^ but not in the UG^18,20^. One potential explanation for the higher cooperation rates in the repeated interactions could also be that individuals learn to anticipate the tit-for-tat sharing behavior of their interaction partners over the course of the game. Indeed, cooperation rates increased slightly for the first repeated interaction across experimental trials. Possible motivators for this learning could be building up trust in the reciprocity as well as a more and more strategic behaviour for improving the personal payoff^38^. In contrast to the latter assumption, cooperation rates remain stable for the second repeated interaction. Further, group affiliation modulates cooperation independent of singular or repeated-trials, which contradicts a mere profit maximizing explanation for the findings behaviour in the presented study. Future studies need to disentangle what exactly individuals were learning in the GSD and which mechanisms enhance cooperation in repeated interactions.

Interestingly, higher psychopathic traits were uniquely associated with lower cooperation in single-trial interactions in the violent offender, but not the control group. This is not consistent with the finding that the absence of cues indicating a possible future interaction predicted non-cooperation in a PD for students scoring high on psychopathic traits^23^. Possible explanations for these diverging results in non-incarcerated individuals may be due to differences in implementation (meet someone in real-life versus repeated interactions in a game) and the use of different psychopathy measures. Further, it is not clear whether psychopathic traits can be used in the same way within non-incarcerated samples as with incarcerated populations and therefore, the previous findings should be interpreted with caution. However, in line with this and other previous findings, the relationship between higher psychopathic traits and reduced cooperation in violent offenders indicates a strategic profit-maximization in single-trial interactions with absence of negative consequences. Future studies are needed to disentangle which specific combination of behavioural, antisocial, and psychopathic characteristics account for these behavioural strategies.

Group affiliation is another important determinant of cooperation and our study is the first to provide empirical evidence for a successful application of a minimal group paradigm in incarcerated individuals. The present data supports previous findings demonstrating an in-group bias for healthy individuals^27^. More importantly, the violent offender group also demonstrated in-group favouritism, revealing no deficits in this key determinant of cooperation. However, contrary to previous studies, psychopathy did not affect the magnitude of the in-group bias^29,30^. Reasons for this discrepancy may be rooted in differences in study samples (students versus incarcerated offenders) as well as different methods to induce group affiliation (in-group and strangers vs. in-group and out-group). Taken together, the fact that violent offenders enhance their cooperative behaviour in response to group affiliation seems to be encouraging. Nevertheless, due to the unspecific manner in which group affiliation was implemented, we have no knowledge which characteristics of the interaction partner may be driving the identification with group members and whether these characteristics deviate in healthy controls and violent offenders. Thus, despite this first evidence indicating similarities between violent offenders and controls in their cooperative behaviour toward in-group members, it remains to be determined which characteristics evoke group affiliation and how this could be used in a therapeutic context.

The current study has several limitation worth noting. First, the current study has been conducted with an exclusively male population; thus, it remains to be determined whether the effects reported in the current study generalize to female and younger externalizing (e.g. children with conduct disorder) populations. Second, the current study is a laboratory investigation that assessed incarcerated inmates. Therefore, it is not entirely clear whether and to what extent the cooperative behaviour assessed in the current study corresponds with real-life behaviour. Prospective study designs are necessary in order to understand how prosocial acts captured in a laboratory setting relate to real-life behaviour. Lastly, we employed a set number of repeated interactions, which may limit the ecological validity. Future studies should introduce more variation in the number of consecutive interactions in order to more closely mimic real-life scenarios.

The present study is one of the first attempts to address the considerable knowledge gap regarding prosocial behaviour in antisocial individuals. The preliminary results indicate generally lower cooperation in violent offenders but intact responding to recipient and situational characteristics that promote cooperation. Despite pessimism regarding psychological interventions for antisocial individuals^5^ and failure to influence cooperation through emotional feedback in individuals from the antisocial spectrum^21,41^, the current findings have potential implications for practice, indicating that these factors can be utilized to promote prosocial behaviour. Future research on modulating psychological factors is needed in antisocial individuals in order to understand the nature of the massive behavioural problems in these populations and to develop treatment strategies to alleviate these symptoms.

## Method

### Participants

Fifty-two incarcerated male violent offenders from cooperating German correctional facilities (Justizvollzugsanstalten Heimsheim, Rottenburg, Hohenasperg) participated in the study. Potential participants were recruited trough advertisement via pamphlets and black boards within the facilities. Inclusion criteria were: 18–65 years, primary conviction for violent crimes and sufficient knowledge of the German language. The facility’s psychological service contacted interested individuals and scheduled the assessments. Clinical and experimental assessments were carried out in designated rooms of the facility by trained psychologists from our research group. Exclusion criteria for the violent offender group were: history of psychotic-spectrum or bipolar disorders (as assessed by clinical interview), primary conviction for drug-related crime. Fifty-two male participants were recruited via advertisements in newspapers and university’s mailing list and served as the control group. Inclusion criteria were: 18–65 years, no self-reported convictions or arrests, no symptoms or full diagnosis of antisocial personality disorder, no history of bipolar or psychosis-spectrum disorder. We matched both groups in terms of years of education through the recruiting process. The study was approved by the Clinical Ethics Committee at the University Hospital Tübingen and was conducted in accordance with the Helsinki Declaration.

### Diagnostic and control measures

Self-reported aggression was assessed with a German version of the Buss-Perry Aggression Questionnaire (BPAQ), an instrument that measures trait aggressiveness with 29 items and the subscales physical aggression, verbal aggression, anger, and hostility. The questionnaire contains statements which participants rate on a 5-point Likert scale ranging from 0 (not at all characteristic for me) to 4 (extremely characteristic of me).

Further, we measured psychopathic traits using the Hare Self-Report Psychopathy Scale III (SRPS). The questionnaire contains 64 items and can be divided into four subscales measuring interpersonal manipulation, callous affect, erratic lifestyle, and criminal tendencies. The items contain statements which are rated on a 5-point Likert scale (1 = disagree strongly to 5 = agree strongly).

To control for IQ related cognitive abilities, the short-version of the *Wiener Matrizen Test* 2 (WMT) was assessed. The WMT is a non-verbal 18-item test derived from Raven’s Progressive Matrices Test that measures problem solving and deductive reasoning. Participants have to match different patterns of matrices to an analogous missing matrix piece by selecting the corresponding part out of eight options.

Current and life-time psychopathology was evaluated with the *Mini International Neuropsychiatric Interview* M.I.N.I.^42,43^ which was administered by trained members of our research group with extensive experience conducting clinical interviews. The M.I.N.I. assesses DSM-IV and ICD-10 criteria for all Axis-I disorders as well as the antisocial personality disorder.

### Experimental task

Cooperation was assessed with a modified version of the sequential GSD paradigm^31,32,40^. We manipulated the number of expected interactions (single-trial interaction vs. repeated-trial interaction) as well as the group affiliation (interaction with in-group vs. out-group member). The dependent variable, cooperative behaviour, was calculated as mean shared MUs for every participant in every condition.

#### Group affiliation

To manipulate group affiliation, we used the well-established minimal group paradigm^33^ by instructing participants before the task that all players including the participants themselves were divided in two groups based on personality characteristics previously assessed in questionnaire measures. The participants were further informed that the group affiliation was indicated by a colour cue (blue or yellow) and that all members of this group were very similar, whereas members of the other group were very different^44^. Subjects were randomly allocated to a group and also to one of the two sets.

#### Stimuli

40 photographs depicting males with neutral facial expression were selected from the Radboud Faces Database^45^ and the Karolinska Directed Emotional Faces Database^46^. Two sets of faces were created, where half of the pictures were marked with a blue background and the other half was marked with a yellow background in one set, the other set contained the same pictures with reverse colouring to guarantee that every face served as an in- or out-group member an equal number of times. Colouring was done with GNU Image Manipulation Program (GIMP). Photographs were presented randomly. A stimulus set compromising additional four male faces with neutral expressions was created for practice trials.

#### Give Some Dilemma task

The participants were informed that they will play a game with a fellow player, who either belonged to the same group (very similar) or to another group (very dissimilar). They received no further information about the identity of the counterpart. Further, they were informed about the opportunity to win MUs, which were later converted into real money (Euro). This additional gain (ranging from 1.80 and 2.10 Euro) to the compensation was implemented to create a more realistic scenario with real-life consequences of cooperative behaviour.

Every trial followed the same pattern: Both players started with ten MUs which they had to divide between themselves and the counterpart at any rate by indicating the number of MUs intended to share via a button press (0–10). Kept MUs counted one fold for the player himself, shared MUs counted twice for the other person. For instance, if a person were to share 4 Mus, the co-player would effectively receive 8 units, while the participant kept the remaining 6. This situation leads to a social dilemma where the individual outcomes are greater to the extent that fewer coins are given away but also both individuals obtain greater outcomes to the extent that they cooperate with each other (give more coins to each other) see^31^.

**Figure 2:**
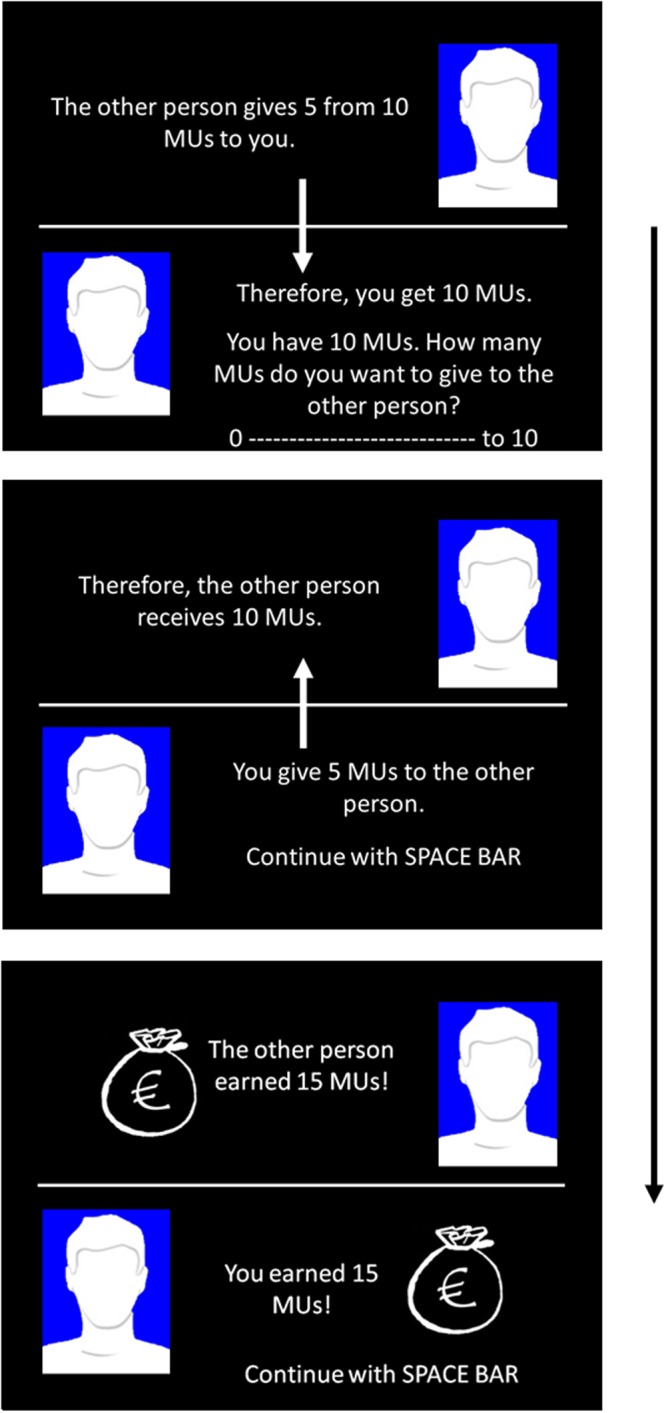
Schematic illustration of a trial in the social dilemma task; MU = monetary unit.

Participants were told that a random algorithm decided that the fellow player always went first. Each trial started with two profile pictures (see Fig. 2): One on the bottom left which was empty, representing the participant, as well as a neutral photo of a male model on the top right. Both profiles were highlighted either blue or yellow to indicate group membership. The screen was divided by a horizontal bar to simulate two sides of a table. Every trial began with a proposition of the fellow player which amounted 5 MUs in 50% of all trials and 4 or 6 Mus in 25% of the trials. This served to ensure fair offers from the fellow player and to enable the participants to reciprocate less, with an equal number of coins, or more coins^40^. Participants received immediate feedback about their gains and subsequently made their offer which also resulted in an instant feedback of total profits for both players.

#### Number of expected interactions

In order to manipulate the number of expected interactions, we designed two conditions of the GSD which were administered in two consecutive blocks with a fixed order. The first block compromised single-trial interactions whereas the second block contained repeated-trial interactions (two interactions). In the latter condition, the first interaction started with the same fair offer as described above, whereas the proposal in the second interaction was analogous to the participants share in the first interaction. This reciprocal cooperation strategy is called tit-for-tat which helps efficiently to establish and retain cooperation^7^.

Participants played 20 trials with 20 different fellow players in the first block and 20 repeated interactions with 20 different fellow players (two trials per fellow player), adding up to a total of 60 trials.

### Procedure

At the beginning of each assessment, participants provided a written informed consent. The diagnostic interview, WMT, and self-report questionnaires (BPAQ, SRPS) were administered before the subjects were introduced to the GSD task. The full instructions for the presented GSD can be found in the Supplementary methods. They completed three practice trials before Block 1 and one before Block 2. All assessments for the control group were carried out in individual laboratory rooms from the University of Tübingen. Violent offenders were assessed in designated rooms within the facility, where none of facility members was present in the room during testing. The experiment was run on a 14.1” HP notebook with a viewing distance of approximately 40 cm. The experiment was programmed and run in Presentation (Version 16.5, Neurobehavioral Systems).

## Electronic supplementary material

Supplementary Information

## Data Availability

The datasets generated during and/or analysed during the current study are available from the corresponding author on request.
