# Supplementary material for: Investigating social-contextual determinants of cooperation in incarcerated violent offenders
Source: Sci Rep. 2018 Nov 21;8:17204. doi: 10.1038/s41598-018-35450-z (PMC6249259; doi:10.1038/s41598-018-35450-z)
Supplement: Supplementary file 1 — Supplementary Information [file 41598_2018_35450_MOESM1_ESM.docx]

Investigating social-contextual determinants of cooperation in incarcerated violent offenders

Jonathan Scheeff^1*^, Aiste Jusyte^2^ and Michael Schönenberg^1^

*^1^ Department of Clinical Psychology and Psychotherapy, University of Tübingen, Germany*

*^2^ LEAD Graduate School & Research Network, University of Tübingen, Germany*

Author Note

*Correspondence concerning this article should be addressed to: Jonathan Scheeff, University of Tübingen, Department of Clinical Psychology and Psychotherapy, Schleichstraße 4, 72076 Tübingen, Germany, Tel.: +49 7071 29-78294, Email: [jonathan.scheeff@uni-tuebingen.de](mailto:jonathan.scheeff@uni-tuebingen.de)

*
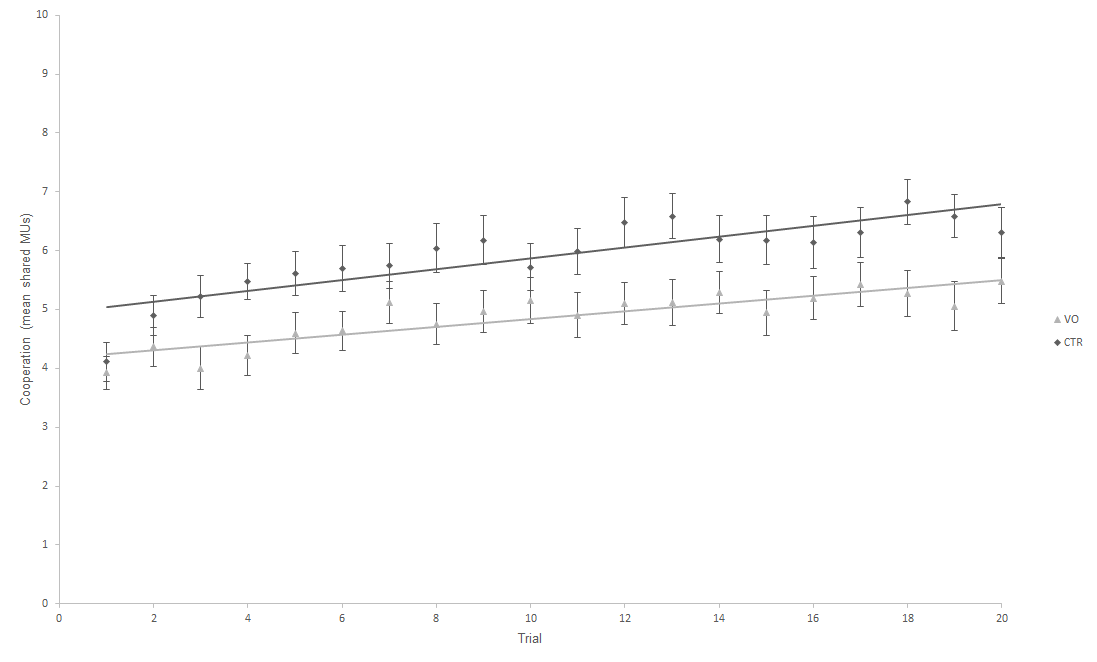
Supplementary Figure S1* Cooperative behaviour in the Give Some Dilemma in the first interaction of the repeated condition for violent offenders (VO, n = 52) and healthy controls (CTL, n = 46). Mean cooperation rates are plotted as a function of shared monetary units (MUs) over the course of the 20 experimental trials. Error bars represent standard error of mean.

*
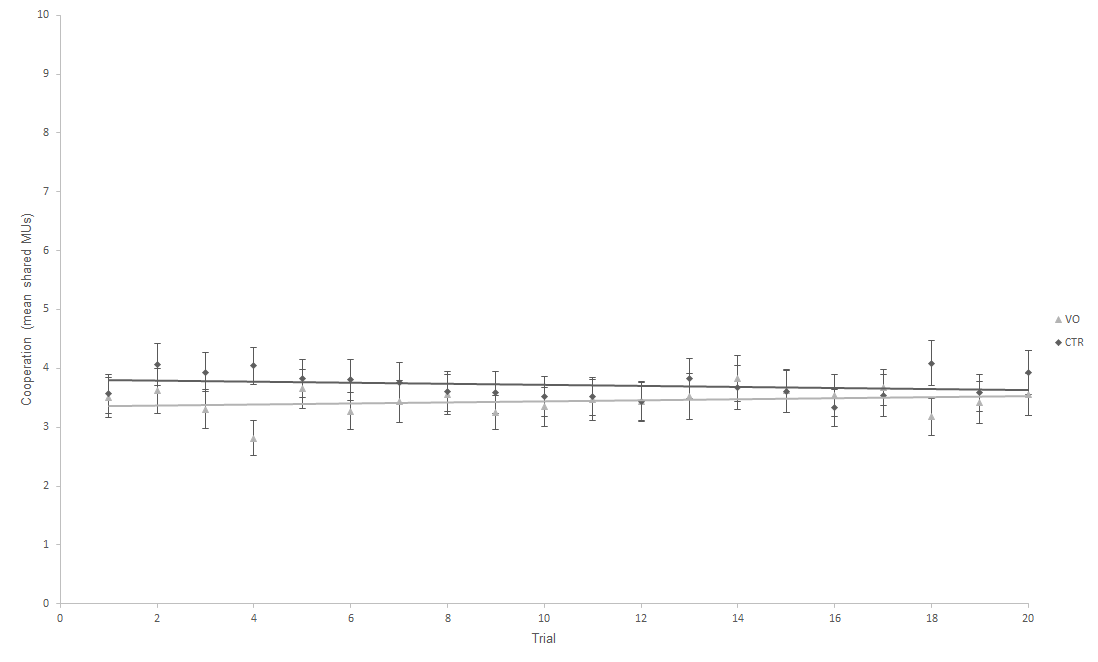
Supplementary Figure S2* Cooperative behaviour in the Give Some Dilemma in the second interaction of the repeated condition for violent offenders (VO, n = 52) and healthy controls (CTL, n = 46). Mean cooperation rates are plotted as a function of shared monetary units (MUs) over the course of the 20 experimental trials. Error bars represent standard error of mean.

**Supplementary methods: Instructions for the Give Some Dilemma**

Welcome! You are going to play a game in which you can acquire monetary units. These monetary units are going to be transferred into a real monetary value which you will get payed additionally at the end of the experiment.

All the players will be assigned into two groups before you actually start with the task: Group blue and group yellow. Whether you belong to group blue or yellow is determined by the computer through your answers in the questionnaire which you answered previously. Your fellow players belong either to your group (which means they are very similar to you) or to the other group (which means they are very dissimilar to you).

Press SPACE in order to learn to which group you belong. The evaluation of you questionnaire shows: You are in group blue.

You will play the game with a different fellow player in each trial. You will receive information about the group affiliation of the other player. You will see the fellow player on a photograph on the top right of the screen. The color indicates whether the fellow player is in group blue (he is very similar to you) or in the other group yellow (he is very dissimilar to you).

Game instructions

You have 10 monetary units. The fellow player has 10 monetary units as well. You can exchange these monetary units with your fellow player. Every monetary unit you hold, has a value of 1 point for yourself and the double value for the fellow player when you share this monetary unit with him. Equally, every monetary unit of your fellow player has a value of 1 for him and the double value for you, if he chooses to share monetary units with you. The computer has determined that the fellow player has the first move and afterwards it’s your turn.

You will receive a feedback about your profit after every trial. Then, a new trial with another fellow player will begin. You start every trial with a budget of 10 new monetary units.

*Three practice trials followed. Control questions after the practice trials:*

To which group did the last fellow player belong?

In which group are you?

What is the color of the other group?

Did you understand the game?

If you have any questions, please call the examiner. Otherwise, you can start the game, when everything is clear.

*Instructions for the second block with repeated interactions:*

You have completed the first part of the experiment. From now on, you are going to play two rounds with every fellow player. Just like before, your fellow player is either in the blue group like you (he is very similar to you) or in the other group, yellow (he is very dissimilar to you). You will play the next trial with a new fellow player after two rounds.

Game instructions

Reminder: You have 10 monetary units. The fellow player has 10 monetary units as well. You can exchange these monetary units between you and your co-player. Every monetary unit you hold, has a value of 1 point for yourself and the double value for the fellow player when you share it.

Equally, every monetary unit of your fellow player has a value of 1 for him and the double value for you, if he shares it with you. The computer decided that the fellow player has the first move and then it’s your turn.
